# Career Adaptability among Migrant Teachers Re-Entering the Labour Market: a Life Course Perspective

**DOI:** 10.1007/s12186-022-09290-y

**Published:** 2022-06-13

**Authors:** Elin Ennerberg, Catarina Economou

**Affiliations:** 1 Department for Society, Culture and Identity, Malmö University, Malmö, Sweden; 2 Department of Culture, Languages and Media, Malmö University, Malmö, Sweden

**Keywords:** Career adaptability, Temporality, Migrants, Life course, Labour market

## Abstract

Amongst the most significant labour market challenges is the integration of migrants and the opportunities for individual migrants to find employment that match their qualifications. The object of this study is to analyse the formal and informal obstacles migrant teachers face when entering the labour market. These obstacles include the formal validation of existing credentials, as well as the needs of local schools, and migrant teachers’ own experiences of the new school system. We have conducted qualitative interviews with school principals, representatives at labour market organizations and authorities and migrant teachers. By analysing these different accounts we argue that the individual career adaptability of migrants also needs to be understood in relation to institutional and organizational constraints. Moreover, by adding a temporal understanding to the integration process, we find that migrants’ own perception of the process and the time-scales of entering work should be accounted for more explicitly in the guidance process.

## Introduction

Re-entering the labour market after forced migration can be a long process for individuals. Such migrants need not only to navigate the challenges related to re-settling but also find routes into a new local labour market. In career counselling and guidance, career adaptability (Savickas & Porfeli, 2012: 662) is often put forward as important for individuals facing challenges related to their career trajectories and to the surrounding social environment. At the same time, other scholars point to the many structural factors facing migrants when attempting to re-enter their former careers (Carlsson & Rooth, 2007: 725).

Tomlinson et al. (2018: 6) discuss how career theories have often focused on “overly agentic” views on individuals’ career choices and opportunities for independently achieving career change. They argue for incorporating life course theory, which takes account of individuals’ life and work transitions with an incorporation of organizational and institutional perspectives of careers. The life course perspective has been used to problematize the more traditional school-to-work transitions, which mainly focused on a male breadwinner route without major interruptions. The life course perspective acknowledges a more varied working life, which, for example, may be interrupted by child care obligations or periods of unemployment. Interruptions in individuals’ careers can also arise through migration.

In this article we attempt to consider both the structural and individual aspects of a certain group of migrant teachers re-entering the labour market in Sweden. This group of teachers with a migrant background have all participated in some form of re-training in order to work in their former profession. The majority have entered Sweden not as labour market migrants but as asylum seekers or family reunification migrants,^1^ and have diverse experience in terms of teaching. These re-training programs can be characterised as a blend of traditional learning activities and vocational training, encompassing an introduction to the Swedish educational system and curriculum (see Ennerberg, 2021). In addition, they can be seen as labour market programs aimed at unemployed individuals (see Ennerberg, 2020). In recent decades there has been a shortage of teachers in Sweden – a shortage projected to increase in the future (SCB, 2017). Utilising the skills and experience of teachers with a migrant background is thus important from a labour market perspective. Moreover, individual teachers can, for example, also bring both subject-specific skills and wider competencies in terms of language skills (Putjata, 2019: 64; Schmidt, 2014).

The purpose of this paper is to analyse the labour market entry of migrant teachers and how they have managed not only career opportunities but also the various structural obstacles they have encountered. The research is needed in order to gain a fuller understanding of how migrant teachers themselves as well as employers and employer representatives can facilitate labour market entry and establishment for this group.

Our research questions are formulated as follows:

- *How do migrant teachers navigate structural and organisational obstacles to be able to find work in Swedish schools?*
- *How do school principals and authority representatives discuss formal and informal obstacles related to re-certification and recruitment?*

The study comprises interview material with individual migrants who have re-entered their former profession in a new context as well as with school principals and caseworkers/representatives of different authorities involved in the recertification process. Because individuals have been chosen for their “successful” entry into the Swedish labour market, a limitation of the study is that we have not considered the stories of those who have been less successful. Consequently, the stories of these migrants cannot be seen as representative of the general group. Nonetheless, by focusing on the trajectories of those individuals who have been able to re-build a career post-migration, we aim to shed some light on, not only the opportunities but also, the possible structural obstacles other individuals might have faced.

## Previous Research

Regarding migrant teachers, previous research shows that they often meet obstacles both in terms of validation of previous skills and education and a de-valuation of skills and competencies (Cho, 2016; Georgi, 2016; Karakasoglu & Dogmus, 2016; Ratkovic & Pietka-Nykaza, 2016). Similarly, studies reveal that local employers often discriminate against workers with a foreign background due to a lack of knowledge of their skills, certification and language skills (Schneider & Lang, 2016: 168).

Teachers with a migrant background often need to manage communications issues related to pronunciation and a different cultural understanding of the national or local education system (Bense, 2014; Fee, 2010; Schmidt, 2014; Bigestans, 2015; Ennerberg, 2020; Marom, 2019). Moreover, teachers with a migration background are sometimes assumed to hold certain specific competencies related to integration and are expected to act as “bridge builders” between the school and pupils and parents with a migration background. These teachers are often seen not only as role models for students but also as extra resources in terms of language and cultural understanding (Georgi, 2016; Schmidt & Janusch, 2016; Schmidt, 2014; Cerna et al., 2019). Some teachers with a migration background, however, prefer not to be labelled as migrant teachers with specific competencies or qualities. Instead, they argue for their right to be recognized as teachers in relation to their professional teacher identity (Bressler & Rotter, 2017; Georgi, 2016; Lander & Sheikh Zaheerali, 2016).

### Conceptual Framework

One of the starting on “life designing points of Savickas’ theory of career construction, or career adaptability, is that individuals today face a far more shifting and insecure labour market than previous generations, who were often able to remain in one or a small number of jobs throughout their working lives (2012). To find their way through the labour market, Savickas and Porfeli (2012: 661) argue that individuals need to focus on “life designing” and managing their identities through constructing a narrative of their working life. While Savickas and others mainly focus on individuals’ “career adaptability” in relation to their careers as a psychological trait (Tokar et al., 2020), other researchers have pointed out that, when considering individuals’ careers, greater attention needs to be paid to institutional structures, organizational constraints and social expectations (Tomlinson et al., 2018: 6). The life course perspective is helpful in understanding biographical patterns, and emphasises the interconnectedness of individuals’ own stories and interpretations of their choices, and the broader structures and limitations in which these take place (Zacher & Froidevaux, 2021). Individuals’ life courses can be seen as “a sequence of socially defined events and roles that the individual enacts over time” (Giele & Elder, 1998: 22). While the sequence of events should be interpreted as individual, i.e. not necessarily following the same sequence, in recent decades, researchers have tended to highlight the increasing insecurity and, to an extent, unpredictability in career trajectories (Ecclestone et al., 2010). That the life course perspective also emphasizes “non-traditional” routes (Bovenberg, 2007), through education and the labour market, can be useful when considering the careers of migrants, who, by definition of their migration, can be seen as having entered a less traditional work trajectory. Moreover, the life course perspective can be useful to consider in relation to the temporality of the migration process. As Giele & Elder argue, individuals often attempt to “tim[e] the events of their lives—in work, education, family behavior, and leisure—so as to make the most of opportunity and suffer the least frustration and failure” (Giele & Elder, 1998:22),

Tomlinson et al. (2018: 6) argue that the life course perspective should emphasize not only individual agency but also institutional environments and organisational settings. Focusing on different levels can highlight the interaction of individuals’ careers and structural constraints and opportunities. In this paper, we take Tomlinson’s argument as a starting point for discussing individuals’ career adaptability in relation to other stakeholders in the process of labour market entry. We do this to shed light on how the organizational settings that individuals have to navigate affect their ability to re-create a career post-migration.

Another theoretical tool that will be used to understand the labour market challenges migrants face is the concept of temporality (see Baas & Yeoh, 2019 for an overview). Compared to more traditional transitions in individuals’ life courses, migrants’ transitions can arguably take on a heightened sense of temporality (Barber & Lem, 2018). For example, migrants applying for asylum “being stuck” and “waiting” can be seen as characterizing features of their lived experience, where their life is effectively on hold (Baas & Yeoh 2019: 163), or at least severely disrupted (Cwerner, 2001). As Barber and Lem (2018: 5) assert, “time’s passing has uniquely personal consequences”. For instance, Robertson (2014: 1928) shows, in the relation to migrants in Australia, that individuals face a “staggered” migration path and great uncertainty regarding whether they will be able to stay in a country permanently due to differing and changing visa regulations on work and migration. Similarly, Griffiths (2014) show how “uncertainty and instability” are crucial for understanding asylum and deportation systems and how these concepts relate to the temporal uncertainties of migration and deportation. While in many cases the temporal aspects of migrants’ time spent waiting are tied up with the period of gaining limited or full residential or citizenship rights, we link the temporal aspects of migration and the heightened sense of temporality to the period after gaining these rights. The temporal aspects of migration in this article will instead be analysed in relation to the post-migration period of attempting to gain access to the labour market.

Consequently, our analysis aims to highlight the different levels impacting individuals’ careers by drawing on the following: 1) the institutional environments exemplified by both education and the labour market in the Swedish welfare state and the discussion of representatives of labour market actors, such as the labour unions and authorities, working with re-certification; 2) the organizational level, represented through the views expressed by potential employers; and 3) the individuals’ own views on their career trajectories.

The issues facing migrant teachers intending to re-enter their former careers are multifaceted and concern different aspects in life. The labour market and educational system that individuals enter can be seen as part of the institutional level that poses certain limitations and possibilities. Similar to other European welfare states (Geddes & Scholten, 2016), individual migrants entering Sweden are expected to participate in labour market activities and language training in order to enter the labour market. Particular to this group is a voluntary opportunity to participate in targeted vocational training in order to facilitate re-certification through an introduction course to teaching for former teachers. This course, however, does not provide migrant teachers with re-certification or any formal qualification. Instead, this process is subject to national regulations of teacher re-certification implemented by the authorities: the National Agency for Education, and the Swedish Council for Higher Education. Moreover, employers’ attitudes regarding migrants’ competencies can form another part of the structural obstacles faced by individuals.

## Methodology

The research was planned to take place at schools through individual qualitative interviews and focus group interviews. Due to the covid-19 pandemic and the restrictions that limited social contact, the plan had to be revised. Thus, almost all individual interviews were conducted digitally. As a result of a previous research project (author, year), we were able to use a number of the same interviewees and gatekeepers to be able to identify potential informants who had previously participated either in the introduction course for teachers or in the more advanced re-certification teaching course (ULV). Emails outlining the aims of the project were sent to potential candidates, with follow-up emails and reminders. Through this sampling process, 19 individual teachers were recruited. Recruiting the school principals was more difficult as many of those contacted declined to participate, citing time pressures and a heavy workload in relation to the pandemic. Ultimately, 9 principals from 4 local municipalities agreed to be interviewed. In addition, representatives from the Public Employment Service, a teacher labour market union, the National Board of Education, The Swedish Council for Higher Education, and the Swedish Association of Local Authorities and Regions were interviewed. These interviews were conducted face-to-face before the Covid-19 pandemic. All interviews were voice recorded and transcribed. Participants consented to the data being collected and received information concerning how to opt out of the research process. As sensitive information was not collected, ethical review was not judged to be necessary for the project. Data has been changed in terms of names and individual participants, and neither schools nor municipalities are named in the article. Furthermore, we have chosen not to refer to participants’ gender. At the start of the data collection, two interviews were conducted and discussed by both of the researchers. Thereafter, the remainder were conducted individually.

Semi-structured interviews are seen as a relevant method to probe informants about questions and gain information about their own experiences on a specific topic (Kvale, 2007). Real-life interviews offer an opportunity not only to find answers to interview questions but also to obtain additional information through body language, small talk and information about the environment, all of which add more informal knowledge to the interview situation (Denscombe, 2014). Digital interviewing offers more in terms of accessibility, thereby facilitating research. However, there are also limitations and challenges, such as forming a more informal rapport with the informant and building trust, as well as showing that the informants’ answers have been understood and keeping the conversation going (Rasche & Platt, 2014). Many of our informants were positive to online participation, in particular the school principals, who found it easier due to the time constraints of their workload. Both the principals and teachers were more than willing to speak about the topic, and, in one sense, the digital platform allowed the informants to quickly engage with the topic without much deviation or small talk. On certain occasions, however, poor internet connection led to mis-communication. In contrast, the face-to-face interviews were often slightly longer and contained more communication on the part of the researcher. While our transcripts of the interviews do not reveal a qualitative difference between online interviews and physical interviews, the tacit understandings and recollections of the interviews taking place in person are stronger when reflecting upon the material. This suggests that online interviewing may be seen as more of a gathering of text and information but may serve as an obstacle to a deeper understanding of interview subjects. The teachers interviewed were asked questions regarding their previous competencies and how their competencies were utilised in their current role. They were also asked to explain their path from arriving in Sweden until their first job and to elaborate on the different types of studies, labour market measures and work experience they had participated in. Questions to principals included: What competencies are important for you when you recruit teachers? And How important are language skills for teachers working at your school? Informants at the different government authorities and interest organisations were asked to elaborate on the process of validation.

After the data collection, both researchers discussed the material and proceeded to develop the different codes and themes. An abductive approach was used, where previous research and theory, to an extent, guided the research and interview questions; however, the themes have also developed through coding and analysis of the data. The abductive approach allowed the researchers to find new aspects of the material through the interviews that could then be related to theoretical understanding. While the researchers wanted to have a flexibility in finding new themes that were not set up as theoretical hypotheses, an inductive approach was not seen as most suitable, as certain theoretical ideas framed the interview questions, for example in relation to guidance and integration in the labour market. The analysis focused on categorising and coding the different themes that emerged from the material through a qualitative content analysis (Patton, 2002).

## Results

In the following sections the results will be discussed in relation to 1) the re-certification process; 2) the organizational pressures and opportunities; and 3) the process of finding a way into work. After presenting the results we will discuss the three different themes in relation to one another under the heading Analysis.

### The Re-Certification Process

Individuals who have completed their teacher training outside of the EU/EES area are required to send their documentation to the National Agency for Education alongside evidence of completed Swedish language courses when applying for a teaching certificate in Sweden. The teaching certificates and other higher education certificates are then sent to The Swedish Council for Higher Education, which ascertains whether the documentation is a valid teacher certificate in the host country and compare it to an equivalent Swedish teacher education.

We compare the different educations. If we say, the starting point is that there is a teacher certification in Sweden, that’s the reason for this process. And in the certification, there are a number of demands. We have legal regulations that are very extensive and detailed. And we follow it as well. (The Swedish Council for Higher Education caseworker).

The comparison with a certified Swedish teacher education is made with what the informants call a “discount”. For example, where a Swedish teacher certificate requires a minimum of 90 higher education (HE) credits of “teacher preparatory” studies – i.e. pedagogics, didactics and practice – this has been reduced to 45 HE credits for migrant teachers.

So, what we look at is the teacher preparatory studies, pedagogics is one part, but it should preferably be pedagogics, didactics, practice. So, if you look globally, the teacher preparatory part is more difficult [to validate] because Sweden is the odd country there. We have an unusually extensive part of the teacher education that involves the teacher preparatory elements. And normally it is 90 HE credits, but it has been reduced. (Respondent, The Swedish Council for Higher Education).

The regulations can thus be applied relatively generously; and in relation to the teacher preparatory studies, teachers with a foreign teaching certificate can “compensate” for the lack of these studies through documented work experience. The specific regulations, however, also entail that teachers gain a very specific diploma or validation upon successful application.

I think that it’s quite unusual from an international perspective. We have the Education Act and also the qualification regulations (*behörighetsförordningen),* which state that in order to get a certification you also get a detailed statement of which subjects, school years and types of schools that you are qualified to teach. And the regulations are very detailed, especially considering they are stated by a national authority, whereas in other countries you might get a teacher certification that is less detailed. (Respondent National Agency for Education).

As the regulations are considered in a relatively detailed manner, participants may find it difficult to estimate the results of the validation process. Moreover, no preliminary decision is given by the authority. One of the interviewees comments on the perceived difficulties facing individuals in terms of predicting the results of the process.

This means that it’s hard for individuals to understand the process, and it’s difficult to get information about the process. It’s hard to weigh up your options […] And it’s also hard for the Public Employment Service – when they meet an individual who says they’ve been a teacher – to know whether this is an individual who, within a reasonable amount of time, can reach a [Swedish] teaching certificate, or whether they won’t. Because if they can’t, it would be more reasonable to recommend an alternative path or fast track to a teaching assistant or some other position that you might take up because you have experience of working in a school. Or maybe that you, long term, might be able to study in higher education as well as validating some of your practical experience (representative, Swedish Association of Local Authorities and Regions).

This representative highlights the insecurity of the validation process and the time aspects in relation to being able to make a decision on whether or not this route is an adequate choice for the individual participant.

### Organizational Pressures and Opportunities

In addition to the formal re-certification process, individual migrants also need to navigate the more subtle organizational requirements of individual employers. The interviews with school representatives (principals) reveal that these requirements vary greatly and, to an extent, disclose the different needs of local schools.

Previous research has suggested that teachers from a migrant background often find work in schools with a high proportion of students with the same background (Collins & Reid, 2012; Fee, 2010). However, our data also discloses that some principals in these schools are more careful when employing staff, pointing mainly to the importance of developing Swedish language skills among their pupils.

Well, the thing is that in my school, of almost 150 pupils, I think I have 3 with Swedish as their first language. So, I need teachers who speak Swedish. It’s very important that teachers speak good or a reasonably correct Swedish. I’m very careful with teachers speaking Swedish and that they try to remind pupils to speak Swedish at breaktimes as well, because most pupils have the same first language. And if they fall back into that and they’re not forced to speak Swedish, then their Swedish won’t develop, and they won’t learn new vocabulary. (Principal 008).

Evidently, in schools where students are struggling with adapting to the Swedish language, teachers’ language competencies are more highly valued than their ability to take on the role of bridge-builders.

However, other principals explicitly refer to utilising migrant teachers as bridge-builders or as role models.

It’s also one of the reasons I thought it [hiring a teacher who had previously participated in the fast-track education] was a good idea. We have many pupils with another mother tongue than Swedish, and I think it’s good to show the pupils different opportunities: that teachers also have different backgrounds and different qualities and skills. (Principal 005).

This principal clearly sees the students’ Swedish language skills as lacking. Moreover, s/he also reflects upon the organizational consequences behind the decision to employ an immigrant teacher.

This very important aspect of security and a calm environment in the classroom is one of the great challenges for this teacher. And it’s easy to be frustrated when this doesn’t work, if the classroom gets unruly then the class teacher can get frustrated. But then we talk about the fact that we have different strengths: s/he has some other great qualities and that their teacher education is very different.[…] So even though s/he has some things to learn and everything doesn’t work perfectly, s/he also has a very tolerant group of colleagues. And the fact that I knew that her colleagues are very good and supportive led to me taking a chance in hiring this teacher. If the circumstances were different, I might not have done it. (Principal 005).

Another school principal in a similar local area also reflects on language issues, but identifies other qualities that experienced migrant teachers bring to the school, as well as their ability to develop language skills during their employment.

Well, we’re in an area where we’re a multicultural school. We have many language groups represented here. And just the obvious point that [migrant teachers] bring other languages and have knowledge of a different culture, it is to speak of something that is incredibly valuable here, both in everyday situations and to help us understand parents and other things. And it’s also the experience they may have in order to compare different education systems, because there are things in other education systems that sometimes may help us to choose some other ways I think. (Principal 006).

Another interviewee refers to both the individual differences among teachers with a migrant background and the importance of finding organizational support for this group to be able to gain access to and stay in the system.

There are migrant teachers that are incredibly appreciated. But if the organisation has to cut back, that person will often be the first to leave. The reason being that it might be a subsidised employment; thus they are not yet hired as regular teachers that manage the whole class room teaching. If you’re the supporting teacher or teacher assistant, then you will have to leave. So it’s not only language related – they haven’t reached a secure place in the organisation yet. […] But I think that we have to work more as municipalities in a structured way to give opportunities, to give support, and then be brave and actually give them a chance. (Principal 009).

This school principal, who works in a smaller municipality, actively works with this group of teachers to provide a more streamlined route into permanent employment. Here, support is provided to individual teachers in different ways and serves both as a form of gatekeeping and providing individuals with the kind of subtle knowledge about informal requirements that is often difficult to acquire independently.

### The Process of Finding a Way to Work

In this section, we will mainly discuss the individuals’ own narratives in relation to the organisational and institutional obstacles discussed above. The individual migrants interviewed consider their routes into the teaching profession from a more or less stable labour market position. Most of them have managed to find a way into the labour market and are now in a position to reflect on the strategic route they took. However, they now see this period as one where both the re-certification and job-seeking processes were somewhat unstable.

One issue brought up by many respondents was that of finding correct information about the re-certification process. Moreover, the bureaucratic process described by the interviewee working in the authority is seen by many interviewees as opaque and daunting. In particular, and similar to the Swedish Association of Local Authorities and Regions’ representative, the uncertainty of how much time needs to be spent on re-training is central to the participants’ stories. For some interviewees, finding reliable information through municipality career services enabled them to better overlook the re-certification process and find the path towards teaching a realistic option.

If I hadn’t had this help, I wouldn’t have been able to reach my goal. We were able to meet a study advisor almost once a week, and she always had new information. And she also arranged for us to meet other migrant teachers who had been fighting to get their re-certification and who had worked as teachers in Sweden. And they came to the classroom and told us their stories. And it always gave us energy. You would say, “If he could do it, why wouldn’t I be able to?” It was clever. (Teacher S).

Others less fortunate to find this type of support and information were disappointed having lost time that could have been spent reaching the goal of teacher validation more quickly.

I actually think that, what are they called, study advisors, that they need better education. Because I went to see them several times, and they never gave my any useful tips. […] They said I had to study Swedish and then complementary studies (ULV). But there are many alternative routes, actually. So I studied Swedish, but there are also other ways. I would have been able to study over the summer, for example. But I didn’t know, they didn’t tell me. They hid information from me. So it’s seven summers that have disappeared from my life, that’s what I feel. I would have been able to get my teacher certification earlier. Do you see what I mean? (Teacher B).

For this participant, there is a clear sense of disappointment in having missed out on important information and even the suspicion of information being hidden. While it seems unlikely that career guidance services would deliberately withhold information, the frustration expressed by this informant may nonetheless be seen as a reflection of the difficulties in overviewing the process. Moreover, it may also signal the need for career services to provide more targeted information to individuals without knowledge of the education system or different authorities.

The sense of temporality as being important for individuals in this position of waiting for re-training is referred to by some of the participants. However, when many of these individuals have reached their desired teaching position, the sense of urgency that is often referred to while in training, is replaced by a sense that time passes quickly and that the opportunities to re-train have to be seen in relation to a longer working-life.

Life goes so fast, so fast. Do you understand? So now, I’ve been about two years in Sweden, but I feel like I just got here. But if I hadn’t done anything in two years, I would think “Oh, what a life, why did I come here?!” So I had to forget these thoughts about why I came to this country, why I didn’t stay. It’s difficult to leave your family and everything. But you have to forget everything and then focus on your goal, that I will be a teacher, maybe after ten years or five years, I will be someone in this society, and influence society in some way. (Teacher B).

Consequently, the difference in time perspectives affects how an individual’s perception of the training course is portrayed. Once established on the labour market, the individual teacher is able to see the re-qualification process as worthwhile, in the sense of it leading to the desired goal. However, during re-qualification, even if the information in the course is portrayed as useful, the course is seen as too time-consuming, with being able to work as a teacher prioritised over the training.

But even if I am a bit annoyed about studying, and studying the same things, the only thing that annoys me is that I have to study something I have already studied. But other than that, I am beginning to kind of enjoy the course, in the sense that it is bringing me back to why I became a teacher in the first place, but also … I am doing right now the course “To Be a Teacher in Sweden”, and as part of that, I am studying “contrastive perspectives in English and Swedish”. And I understand a little bit more, and now I can really compare with my background as a teacher and my … the background I am building as a teacher in Sweden, and I can see the different roles. And I can see … then I can see what can I bring to the table, what should I get out of my … So, it is okay. It is good. If I could do this in six months, that would be lovely. Not in two years. (Teacher A).

For this participant, the time it takes to complete the course overshadows the learning perspectives. Moreover, this teacher considers the process as unjust in light of his/her previous education and experience, which is here seen as overlooked. In this sense, the teacher expresses a feeling of discrimination that is tied to what is perceived as unjust expectations in relation to validation of previous educational skills, as well as a devaluation of the practical teaching skills.

## Analysis

By considering the perspectives of caseworkers, school principals and individual migrant teachers, we can trace the different levels of the institutional and organisational contexts that influence the individuals’ career adaptability. Rather than being able to independently craft out a new career trajectory, individuals are affected by a re-certification process and employment process where other considerations are taken into account. In this sense, our findings show how the institutional level of higher education credentials, national teaching regulations, organisational levels of local municipalities, and school principals, influence the individuals’ work trajectories and can serve as obstacles in their careers. Rather than being negotiable on the individual level, these obstacles can also severely limit career changes in the life course when attempting to re-enter and re-create a professional career (cf Tomlinson et al., 2018). The values and considerations, shaping the re-certification, mix formal requirements of comparing the Swedish teacher education to migrants’ own qualifications with a more informal part of the process where work experience is taken into account. From the teachers’ own perspectives, however, the process often appears obscure. “Career adaptability” in this case is often limited to attempting to find ways to navigate the system. However, individuals’ own sense of difficulties in being “stuck” in this process of waiting for their certification can influence their choices of whether or not it is worth re-entering their career as a teacher. In contrast, the individuals who through career guidance have received information about the process and can relate it to a particular, and set, time frame ultimately seem to have more faith in the process working to their advantage. In line with previous literature, temporality here seems important, particularly in relation to being able to re-enter work in line with individuals’ preferred life course, as individuals experience “time’s passing” (Barber & Lem, 2018: 5) urgently while waiting to re-enter their preferred work trajectory. Thus, clearly understanding the time involved in re-training or re-certification enables individuals to regain control of their time, rather than experience what Cwerner (2001) sees as “socially disrupted time”. We have shown that the temporality of migration can be seen not only in relation to earlier points in the migration process, but have continued to mark the individuals’ experiences of transitioning into education and work.

In relation to the labour market, finding schools that give the individual the opportunity to utilise one’s skills is a second step: one that requires the individuals to engage with organisational constraints. Despite formal qualifications, school principals’ local needs – as well as hesitancies of whether migrant teachers’ formal qualifications match the national or local teaching competencies – serve as a further potential obstacle. The individual migrant teachers’ own engagement with the educational opportunities seem to facilitate their adaptation to a somewhat new teaching role. Here, attending the course and finding work experience, as well as formal knowledge of the system, can thus be seen as a bridge to local labour market knowledge as required by the school principals. At the same time, some participants see these courses as a type of “time trap” where the temporal aspects of being stuck in a lower labour market position, in order to validate knowledge the individuals already have, overshadow the more positive aspects of the education. Compared to Robertson’s (2014: 1928) concept of “staggered” migration trajectories, individuals here seem to experience “staggered labour market entry”, which nonetheless relates to their position as migrants. For individuals who have secured a permanent position and can look back on the process, time has taken on a very different quality. From this vantage point, the temporal aspects of going through the process into employment are instead seen as time well spent, rather than as urgently passing. From this more secure position, the temporal aspects are downplayed, or instead utilised as a way to consider future career steps that have opened up new opportunities. The teachers have attempted to restructure their life course in relation to the new opportunity structures that they have encountered in terms of the new demands in relation to their careers. At the same time, the “timing” (cf Giele & Elder, 1998: 22) of events that fit these work opportunities into other aspects of life, such as childcare or family life, are also highlighted in the respondents’ stories. Finding new ways into the labour marketare also, in the respondents’ interrupted life courses, considered in relation to other social and individual demands. Concomitantly, attempting to reach one’s goals while avoiding pain and suffering, may also mean that some individuals accept lower level labour market positions or refrain from taking the route into teaching, thus reshaping their life course towards a different career trajectory. For the individual teachers in our study who have been successful in terms of staying on their previous career path, time spent waiting for labour market entry has instead become an opportunity for being able to plan and predict one’s work trajectory in relation to the structures and limitations that the teachers have encountered during the process.

## Conclusion

In the results section of the article, we discussed the perspectives of teachers, school principals/employers and organisational representatives, to show the different levels of career adaptability and how the institutional and organisational levels intersect with participants’ own narratives of how they adapt to a new labour market.

A central part of the institutional framework confronting migrant teachers is the re-certification process. As we saw in the first part of the results section, this bureaucratic process is portrayed by representatives as attempting to contain a degree of flexibility, such as allowing for individuals to replace certain formalised educational competencies with work experience as teachers. At the same time, other representatives point to the difficulties in understanding and predicting the outcome of the process, which serves as a potential deterrent for individuals in a precarious labour market position. The individual teachers facing this process in a similar way underline the re-certification as difficult to understand, thereby leading to great uncertainty. This is in line with previous research that emphasises the bureaucratic aspects of the process (Schmidt, 2014). However, we also saw that, for those who have received correct information by guidance counsellors or others, the ability to overview the process makes it possible to evaluate the time spent on re-training. While the teacher re-certification process aims to protect the teaching profession and educational system, the school principals represent the organisational interests of individual schools that set up their own boundaries. Here, some of their views are that the formal qualifications are not always reliable when employing migrant teachers. This implies a potential of discrimination based on language skills or prior qualification, which has also been noted in other international research studies (cf Bense, 2014; Fee, 2010). However, in our study there were also some school principals who chose to employ individual teachers, despite what they see as a lack of language skills or the more abstract idea of what a “good teacher” means in the Swedish or local school context. In these cases, school principals see their efforts to employ teachers with a migrant background from a perspective of a societal responsibility or representativity in relation to students with a migrant background. In other cases, they argue that the already pressured school system does not always allow for the risk implied in employing individuals who have not yet taught in the Swedish context. The local situation of the school generally played a large part in principals’ discussions - a finding that can be useful, on both a policy and a research level, as there seems to be a gap in relation to national goals and initiatives to facilitate migrant teachers’ entry into school with inadequate organizational support and services locally.

The perceived risk put forward by employers has the effect of creating uncertainty for individual teachers, who instead of gaining permanent employment as teachers may be required to take on lower level positions or temporary employment in order to prove their skills. Here, career adaptability means the forced acceptance of re-training and a prolonged time in insecure employment(s). Career adaptability as expressed by the participants thus often in practice entails further re-training in order to adjust to organisational and institutional demands. In many ways, the participants try to adapt to the different regulations and demands of employers. However, the need for increased adaptability is often accompanied by a strong uncertainty of whether or not the chosen path will actually lead to employment in the field of teaching. Individuals with a stronger sense of having been given the correct information see the process of adapting to the needs of the labour market more clearly, given transition points where the end goal is in sight. One finding that surprised us in the study was that learning about the process, and the education system and labour market, was also used to continue searching for opportunities beyond the teaching position, such as applying for other university courses or in other ways building up skills.

## Footnotes

1 While we recognize that the categorisation of migrants is far from unproblematic, we, nonetheless, here refer to the status given to the individuals as this is relevant for their participation in some of the labour market training programs referred to in the article text.
